# Functional and proteomic characterization of *Phytophthora nicotianae* extracellular vesicles and putative cargo-sorting signals in filamentous pathogens

**DOI:** 10.3389/fpls.2026.1899248

**Published:** 2026-09-11

**Authors:** Celiwe I. Nxumalo, Brenda C. Salasini, Silindile Maphosa, Lucy N. Moleleki

**Affiliations:** Department of Biochemistry, Genetics and Microbiology; Centre for Microbial Ecology and Genomics, University of Pretoria, Pretoria, South Africa

**Keywords:** extracellular vesicles (EVs), filamentous pathogens, KFERQ-like motifs, *Phytophthora nicotianae*, plant-pathogen interactions

## Abstract

Extracellular vesicles (EVs) are lipid-bound structures that transport bioactive molecules and are increasingly recognized as key mediators of plant-pathogen interactions. Although EV secretion has been reported in filamentous pathogens, their roles in plant immunity, microbial interactions and cargo-sorting mechanisms remain poorly understood. This study aimed to characterize the functional roles and composition of EVs produced by *P. nicotianae*, and to investigate shared EV-associated protein categories and putative cargo-sorting signals, including KFERQ-like (Lys-Phe-Glu-Arg-Gln) motifs, across filamentous pathogens. EVs were isolated and characterized using transmission electron microscopy (TEM) and Nanoparticle tracking analysis (NTA). TEM confirmed the presence of membrane-bound vesicles with both single- and double-membrane morphologies, while NTA demonstrated a reproducible and heterogeneous particle population. Functional assays demonstrated that *P. nicotianae* EVs elicit immune-associated responses in *Nicotiana benthamiana*, including cell death, ROS accumulation, and callose deposition. In addition, the EVs exhibited antimicrobial activity against five soil-associated bacterial isolates. Proteomic analysis identified 2,324 proteins, revealing a diverse EV cargo enriched in metabolic enzymes, protein-modifying enzymes, and transporters, along with virulence-associated proteins. Comparative analysis across selected filamentous pathogens identified shared EV-associated protein categories, including ATP synthase subunits, elongation factors, and heat shock protein 70 (HSP70), present across all examined species. Notably, approximately 78% of EV-associated proteins from nine filamentous pathogens contained putative KFERQ-like motifs. Collectively, these findings show that *P. nicotianae* secretes structurally heterogeneous EV-like particles with biological activity in plant and bacterial assays, and suggest potential roles in host interaction, microbial competition, and putative conserved features that may be involved in cargo loading.

## Introduction

Extracellular vesicles are lipid bilayer-bound structures released by cells into the extracellular environment (Gregory and Rimmer, 2023). These vesicles are produced by both prokaryotic and eukaryotic organisms under normal physiological conditions as well as in response to stress (Abramowicz et al., 2019; Gill et al., 2019; Kalluri and LeBleu, 2020; Chiaradia et al., 2021). In eukaryotes, EVs are classified into apoptotic bodies, microvesicles, and exosomes based on their size and biogenesis. Apoptotic bodies are generated through membrane blebbing during programmed cell death, microvesicles arise from the outward budding of the plasma membrane, and exosomes originate from multivesicular bodies that fuse with the plasma membrane to release vesicles into the extracellular space (Kang et al., 2021; Chen and Yang, 2024; Woith et al., 2021). EVs carry a diverse range of bioactive molecules, including lipids, proteins, nucleic acids, and metabolites (Solé et al., 2015; Yáñez-Mó et al., 2015; Zaborowski et al., 2015; McMillan et al., 2021; Woith et al., 2021; Hao et al., 2024; Sheng et al., 2025). They have been extensively characterized in bacterial and mammalian systems, where they play critical roles in intercellular communication, the maintenance of cellular homeostasis, pathogenesis and microbe-interactions (Yoon et al., 2014; Kim et al., 2015; Bose et al., 2020; Pirolli et al., 2021; Munoz et al., 2022).

Extracellular vesicles have been reported in a range of plant fungal species, including *Fusarium oxysporum f.* sp. *vasinfectum*, *F. graminearum*, *Colletotrichum higginsianum*, *Botrytis cinerea*, and *Ascochyta rabiei*, as well as in oomycetes such as *Phytophthora capsici*, *P. sojae*, and *P. infestans* (Bleackley et al., 2020, Garcia-Ceron et al., 2021; Fang et al., 2021; Rutter et al., 2022; De Vallée et al., 2023; Zhu et al., 2023; Breen et al., 2025; Ghaheri et al., 2025). Proteomic analyses have revealed that these EVs contain proteins with diverse functional roles, including metabolic enzymes, components involved in RNA metabolism, translation, membrane trafficking proteins, and various transporters. Notably, EVs are enriched in virulence-associated molecules such as pathogen-associated molecular patterns (PAMPs), cell wall-degrading enzymes (CWDEs), and both apoplastic and cytoplasmic effectors. Furthermore, proteomic studies in *Phytophthora* species have led to the identification of EV-associated marker proteins. For example, in *P. sojae*, the tetraspanin family proteins PsTET1 and PsTET3 have been identified as EV markers, while in *P. infestans*, MARVEL domain proteins PiMDP1 and PiMDP2 have similarly been proposed as EV-associated markers (Zhu et al., 2023; Breen et al., 2025). However, despite these advances in compositional analyses, the functional roles of EVs in filamentous pathogens remain poorly understood. Only a limited number of studies have demonstrated direct biological activity, with EVs from *F. oxysporum f.* sp. *vasinfectum*, *P. sojae*, and *P. capsici* shown to induce plant cell death (Bleackley et al., 2020; Fang et al., 2021; Zhu et al., 2023).

A major unresolved question in EV biology in filamentous plant pathogens concerns the mechanisms that govern selective cargo loading. In mammalian systems, KFERQ-like motifs have been implicated in the selective incorporation of a subset of soluble proteins into specific exosomal populations through a chaperone-associated pathway involving heat shock cognate protein 70 (HSC70/HSPA8) and lysosome-associated membrane protein 2A (LAMP2A) (Ferreira et al., 2022; Xu et al., 2025). KFERQ-like motifs are defined by physicochemical composition rather than strict sequence conservation and typically consist of a pentapeptide containing one glutamine residue, one negatively charged residue, one or two positively charged residues, and one or two hydrophobic residues. These motifs occur as canonical forms or can be generated through post-translational modifications, including acetylation and phosphorylation (Schnebert et al., 2022). Functionally, KFERQ-like motifs are best established as recognition signals in chaperone-mediated autophagy, where they facilitate selective substrate recognition by HSC70/HSPA8. More recent studies indicate that related recognition principles may also contribute to selective EV cargo-sorting in mammalian cells (Ferreira et al., 2022). Notably, approximately 62% of mammalian exosomal proteins have been reported to contain KFERQ-like motifs, supporting a potential association between these motifs and EV cargo composition, although motif presence alone does not demonstrate functional sorting activity (Ferreira et al., 2022). Whether analogous motif-dependent cargo selection mechanisms operate in filamentous plant pathogens remains unresolved, particularly given the distinct secretory organization, polarized hyphal growth, and infection-associated developmental structures that characterize these organisms.

*P. nicotianae* is a highly destructive oomycete pathogen capable of infecting over 255 plant species, including economically important crops such as potato, tomato and citrus (Cline et al., 2008; Wang et al., 2024). Given the limited understanding of EV function and cargo-sorting mechanisms in filamentous pathogens, this study investigates the functional roles and protein composition of EVs in *P. nicotianae*, with particular emphasis on identifying shared EV-associated protein categories and evaluating the prevalence of putative cargo-sorting signals, including KFERQ-like motifs, across filamentous pathogens.

## Materials and methods

### Strain and culture conditions

*Phytophthora nicotianae* INRA 310 was cultured on V8 juice agar plates (10% V8 juice, 1.4 g CaCO_3_, 15 g agar, distilled water to 1 L) and incubated at 25 °C for 3 days. V8 juice agar was used as the routine maintenance medium to obtain actively growing mycelia for inoculation into liquid culture (Erwin and Ribeiro, 1996). Fifteen agar plugs (5 mm diameter) were excised from the actively growing colony margin and transferred into 1 L potato dextrose broth (PDB) supplemented with *N. benthamiana* leaf extract. Uninoculated PDB supplemented with *N. benthamiana* leaf extract was subjected to the same EV-isolation procedure and used as the mock (negative) control. The leaf extract was included to provide plant-derived biochemical components during pathogen growth using a host-supplemented liquid culture approach similar to that described by Ghaheri et al. (2025). Cultures were incubated at 25 °C with shaking at 121 rpm. Cultures were harvested at time points appropriate for the respective downstream analyses. Four-day cultures were used for scanning electron microscopy, whereas eight-day liquid cultures were used for EV isolation to allow the accumulation of EVs in the conditioned culture medium before purification.

### Scanning electron microscopy

Scanning electron microscopy was performed to examine the surface morphology of *P. nicotianae* hyphae and to examine the hyphal surface for vesicle-like extracellular structures before EV isolation. After 4 days of growth, 5 mL of culture was harvested. Mycelia were pelleted by centrifugation at 3,000 rpm for 3 min, washed three times with 0.1 M phosphate buffer (pH 7.4), and fixed in 2.5% (v/v) glutaraldehyde for 30 min at room temperature. Post-fixation was performed using 1% (w/v) osmium tetroxide for 30 min. Samples were dehydrated through a graded ethanol series (30%, 50%, 70%, 90%, and 100%), with each step lasting 10 min; absolute ethanol was applied three times (10, 10, and 40 min). Samples were then incubated in a 1:1 mixture of hexamethyldisilazane (HMDS): ethanol for 1 h, repeated twice, followed by incubation in 100% HMDS. Samples were air-dried, mounted, carbon-coated, and imaged using a Zeiss Crossbeam 540 Field Emission-Scanning Electron Microscope (Zeiss, Germany) at 1.0 kV.

### Isolation of EVs from *P. nicotianae*

The EVs and uninoculated PDB mock were prepared from eight-day-old liquid cultures grown in PDB supplemented with *N. benthamiana* leaf extract at 25 °C with shaking at 121 rpm, as described above. Mycelia were removed by filtration through double layer micro-cloth. The filtrate was subjected to sequential centrifugation at 4,000 × *g* for 15 min and 15,000 × *g* for 15 min at 4 °C to remove cellular debris. The culture supernatant was first passed through a 0.22 µm membrane filter to remove *P. nicotianae* and other cellular material. The resulting filtrate was subsequently inoculated into fresh PDB and incubated for 4 days under conditions suitable for *P. nicotianae* growth to confirm the absence of viable *P. nicotianae* in the filtrate. Following confirmation of no detectable fungal growth, the cell-free filtrate was concentrated approximately 20-fold using a 50 kDa amnicon tubes. The concentrated filtrate was subsequently passed through a 0.22 µm membrane filter to minimize the possibility of microbial contamination. The EVs and uninoculated PDB mock preparations were subsequently ultracentrifuged at 100,000 × *g* for 1 h at 4 °C. The samples were washed twice with phosphate-buffered saline (PBS) using the same ultracentrifugation conditions, resuspended in PBS, and stored at −80 °C until further analysis.

### Transmission electron microscopy

EV morphology was examined by negative-staining TEM. EV suspensions were adsorbed onto carbon-coated copper grids for 5 min, excess liquid was removed, and grids were stained with 1% (w/v) uranyl acetate for 3 min. Grids were air-dried and visualized using a JEOL JEM-2100F transmission electron microscope (JEOL Ltd., Japan). The uninoculated PDB mock control was examined in parallel as a negative control.

### Nanoparticle tracking analysis and protein quantification

Size distribution and particle concentration in the EV and uninoculated PDB mock preparations were analyzed using a NanoSight NTA system (Malvern Panalytical, UK) with NTA software version 3.3. Three independent biological replicates were analyzed. For each replicate, 1.5 mL of EV or uninoculated PDB mock preparation was analyzed by recording 30 s videos at 20–22 °C. Camera settings and detection thresholds were standardized across all samples. PBS was analyzed as a blank control. Protein concentrations of the EV and uninoculated PDB mock preparations were determined using the Bradford assay (Bio-Rad, USA), according to the manufacturer’s instructions, with bovine serum albumin (BSA; Sigma-Aldrich, USA) used as the protein standard.

### Inoculation of *N. benthamiana* leaves with EVs

EV and uninoculated PDB mock control preparations were washed twice with PBS by ultracentrifugation at 100,000 × g for 1 h at 4 °C and resuspended in PBS. *N. benthamiana* leaves were treated under five conditions: (i) PBS control, (ii) uninoculated PDB mock, (iii) EVs (1 x 10^9^ particles/mL), (iv) EVs + *P. nicotianae* mycelial plug and (v) mycelial plug alone. Treatments were performed on detached leaves, which were placed in containers and incubated under controlled conditions and monitored for symptom development. All experiments were performed using three independent biological replicates, with each biological replicate consisting of four technical replicates. Lesion size was measured for each treatment, and the data are presented as the mean ± standard deviation (SD). Statistical analyses were performed using GraphPad Prism. Differences among treatments were analyzed using one-way analysis of variance (ANOVA) with statistical significance set at *P* < 0.05.

### Detection of reactive oxygen species and callose deposition

Reactive oxygen species accumulation and callose deposition in *N. benthamiana* leaves were assessed following treatment with PBS, uninoculated PDB mock, EVs, mycelia and EVs + mycelia. Leaves were harvested 24 h post-treatment. For ROS detection, harvested leaves were stained overnight in the dark with 1 mg/mL 3,3’-diaminobenzidine (DAB-HCl, pH 3.8), followed by de-staining in 96% (v/v) ethanol (Daudi and O’Brien, 2012). ROS accumulation was examined under a light microscope at 10× magnification and ROS levels were quantified using ImageJ software (Schneider et al., 2012). For callose deposition analysis, chlorophyll was removed from harvested leaves using 95% (v/v) ethanol. The cleared leaves were then stained with 0.01% (w/v) aniline blue prepared in 150 mM K_2_HPO_4_ (pH 9.5) for 30 min. Callose deposits were visualized using a fluorescence microscope equipped with a DAPI filter (excitation: 370 nm; emission: 509 nm; Leica Microsystems, Wetzlar, Germany) (Schenk and Schikora, 2015) and quantified using ImageJ software. Three independent biological replicates were analyzed, with four infiltrated leaves per treatment. Five randomly selected fields of view were captured from each leaf using identical microscope settings. Background fluorescence was subtracted before measuring signal intensity. Data are presented as mean ± SD. Statistical significance was determined using one-way ANOVA followed by Tukey’s multiple comparison test (*P* < 0.05).

### Antimicrobial activity of EVs

The antibacterial activity of EVs was evaluated against bacterial isolates obtained from crop rotation soil in the Free State province, South Africa. This field previously experienced severe *Pectobacterium*-induced soft rot in potato, followed by crop rotation practices that promoted the re-establishment of soil microbial communities. From this soil, several bacterial strains were isolated and identified with16S rRNA gene sequencing. The identified bacterial strains included *B. thuringiensis*, *Lysinibacillus pakistanensis*, *Peribacillus frigoritolerans*, *Priestia aryabhattai* and *P. megaterium*. Antibacterial activity was assessed using an agar well diffusion assay. Bacterial cultures were grown in LB broth to an optical density (OD_600_) of 0.5, and 100 µL of each culture was evenly spread onto LB agar plates. Wells with a diameter of 6 mm were loaded with 50 µL of PBS and uninoculated PDB mock, gentamicin (25 µg/mL) as the positive control, and EVs at a concentration of 1 x10^9^ particles/mL. Plates were incubated at 28 °C for 24 h, after which zones of inhibition were measured. The diameter of the inhibition zone surrounding each well was measured in millimeters (mm). All experiments were performed using three independent biological replicates, with four technical replicates per treatment.

### Protein extraction and digestion

Extracellular vesicle proteins from three biological replicates were extracted according to the protocol provided by BGI Tech Solutions Co., Ltd. (Hong Kong, China). Freeze-dried EV samples were resuspended in SDS-free protein lysis buffer supplemented with 1× protease inhibitor cocktail. Dithiothreitol (DTT) was added to a final concentration of 10 mM and samples were homogenized using an automatic grinding instrument. The homogenates were centrifuged at 25,000 × g for 15 min at 4 °C and the supernatants were collected. Samples were further reduced with 10 mM DTT at 37 °C for 30 min, followed by alkylation with 55 mM iodoacetamide (IAM) in the dark for 45 min at room temperature. Proteins were precipitated with five volumes of pre-chilled acetone at −20 °C for 2 h and centrifuged at 25,000 × g for 15 min at 4 °C. The resulting protein pellets were air-dried, resuspended in SDS-free protein lysis buffer, and centrifuged again at 25,000 × g for 15 min at 4 °C. The final supernatants containing the extracted proteins were collected for downstream proteomic analyses.

For enzymatic digestion, 50 μg of protein from each sample was loaded into a 10 kDa ultrafiltration tube and adjusted to equal volumes using the corresponding dissolution buffer. Samples were centrifuged at 12,000 × g for 20 min at 20 °C, followed by three washes with 100 μL of 50 mM ammonium bicarbonate (NH_4_HCO_3_). Trypsin was added at a 1:20 (w/w) trypsin-to-protein ratio and samples were incubated overnight at 37 °C. Following digestion, peptides were collected by centrifugation at 12,000 × g for 20 min at 20 °C. An additional wash with 100 μL of 50 mM NH_4_HCO_3_ was performed to maximize peptide recovery. The collected peptide digests were freeze-dried prior to LC-MS/MS analysis.

### Liquid chromatography-mass spectrometry analysis

Freeze-dried peptide samples were reconstituted in mobile phase A (2% acetonitrile [ACN], 0.1% formic acid [FA]) and centrifuged at 20,000 × g for 10 min. The resulting supernatants were injected into a Thermo Fisher Scientific Thermo UltiMate 3000 UHPLC system for peptide separation. Peptides were first enriched and desalted using a trap column and subsequently separated on a self-packed C18 analytical column (150 μm inner diameter, 1.8 μm particle size, ~35 cm length) at a flow rate of 500 nL/min. Mobile phase A consisted of 2% ACN with 0.1% FA, while mobile phase B consisted of 98% ACN with 0.1% FA. The gradient elution program was as follows: 0–5 min, 5% B; 5–95 min, 5-25% B; 95–105 min, 25-35% B; 105–110 min, 35-80% B; 110–115 min, 80% B; and 115.5–120 min, 5% B. Separated peptides were ionized using a nano-electrospray ionization (nanoESI) source and analyzed using a Thermo Fisher Scientific Q-Exactive HFX tandem mass spectrometer operating in data-independent acquisition (DIA) mode. The ion source voltage was set to 2 kV, with a full MS scan range of 400-1,250 m/z at a resolution of 120,000. The maximum ion injection time was set to 50 ms. The mass range was divided into 45 variable isolation windows for DIA acquisition. Fragmentation was performed using higher-energy collisional dissociation (HCD), and fragment ions were detected in the Orbitrap at a resolution of 30,000. Normalized collision energies of 22.5, 25 and 27.5 were applied, and the automatic gain control (AGC) target was set to 1 × 10^6^.

### Data-independent acquisition, processing and protein identification

Data-independent acquisition raw files were processed using the FragPipe computational platform (version 20.0), with peptide-spectrum matching and protein identification performed using the MSFragger search engine (version 3.2) (Kong et al., 2017) against the UniProt *Phytophthora nicotianae* INRA-310 protein sequence database, downloaded from UniProt on 16 March 2025. Trypsin was specified as the proteolytic enzyme, allowing a maximum of two missed cleavages. Carbamidomethylation of cysteine residues was specified as a fixed modification, whereas oxidation of methionine, protein N-terminal acetylation, pyro-glutamate formation from N-terminal glutamine, and deamidation of asparagine and glutamine residues were specified as variable modifications, with a maximum of three variable modifications permitted per peptide. A minimum peptide length of seven amino acids was required. Protein inference was performed using ProteinProphet (Nesvizhskii et al., 2003), and only protein groups supported by at least two unique peptides were retained for downstream quantitative analysis. False discovery rates (FDRs) were controlled at 1% at both the peptide-spectrum match (PSM) and protein levels. Label-free protein quantification was performed using IonQuant (Yu et al., 2020) based on extracted ion chromatogram (XIC) intensities.

### Sequence analysis and annotations

Protein sequences corresponding to EV-associated proteins were retrieved from the UniProt database and used for downstream functional annotation analyses. Gene Ontology (GO) enrichment analysis was performed using the PANTHER classification system (Mi et al., 2005; Gene Ontology Consortium, 2019) to evaluate overrepresented molecular function categories within the EV proteome relative to the reference dataset. Statistical significance of enrichment was assessed using Fisher’s exact test. Subcellular localization predictions were conducted using both WoLF PSORT and DeepLoc-2.1 (Horton et al., 2007; Ødum et al., 2024) to improve prediction robustness across extracellular, membrane-associated and intracellular protein classes. Effector-like properties of EV-associated proteins were assessed using EffectorP 3.0, which predicts candidate apoplastic and cytoplasmic effectors in fungi and oomycetes based on machine-learning approaches trained on experimentally validated effectors (Sperschneider et al., 2018; Sperschneider and Dodds, 2022). To evaluate secretion-associated features, N-terminal signal peptides were predicted using SignalP-6.0 (Teufel et al., 2022), while transmembrane helices were identified using TMHMM v2.0c (Krogh et al., 2001). These analyses were used to distinguish proteins associated with canonical secretion pathways from proteins potentially associated with non-canonical extracellular export mechanisms.

### Comparative analysis of EV-associated protein categories among filamentous pathogens

Published EV proteome datasets from fungal and oomycete pathogens were collated for comparative analysis with *P. nicotianae*. The fungal species included *F. oxysporum f.* sp. *vasinfectum*, *F. graminearum*, *C. higginsianum*, *B. cinerea*, and *A. rabiei*, whereas the oomycete species included *P. capsici*, *P. sojae*, and *P. infestans* (Bleackley et al., 2020; Garcia-Ceron et al., 2021; Fang et al., 2021; Rutter et al., 2022; De Vallée et al., 2023; Zhu et al., 2023; Breen et al., 2025; Ghaheri et al., 2025). These species were selected based on the availability of published EV proteome datasets and their agricultural importance. Protein annotations reported in the published EV proteomes were manually reviewed and standardized by consolidating synonymous protein names and equivalent functional annotations into common protein categories. Comparisons among species were therefore based on these standardized protein categories rather than exact protein names. A binary presence-absence matrix was constructed by assigning each protein category a value of 1 (present) or 0 (absent) for each species. Pairwise Jaccard similarity coefficients were calculated from the binary matrix as the proportion of shared protein categories relative to the total number of protein categories present in either species. The resulting similarity matrix was visualized as a categorical heatmap to illustrate conserved and lineage-specific EV-associated proteins among species.

### Identification and classification of KFERQ-like motifs

EV-associated proteins from *F. oxysporum f.* sp. *vasinfectum, F. graminearum, C. higginsianum*, *B. cinerea*, *A. rabiei*, *P. capsici*, *P. nicotianae* and *P. infestans* were screened for KFERQ-like motifs using computational criteria adapted from established chaperone-mediated autophagy (CMA) definitions (Dice, 1990; Kirchner et al., 2019; Schnebert et al., 2022). Conserved RxLR (Arg-X-Leu-Arg) effector repertoires from 36 *Phytophthora* species were obtained from Salasini et al. (2026) and analyzed using the same analytical framework. KFERQ-like motifs were identified on the basis of physicochemical composition rather than strict sequence identity, consistent with canonical CMA-targeting principles. Motifs were defined as pentapeptide sequences containing one glutamine residue (Q), one negatively charged residue (D or E), one or two positively charged residues (K or R), and one or two hydrophobic residues (F, I, L, V or Y) (Dice, 1990; Kaushik and Cuervo, 2018). Motif detection was performed using a sliding-window approach implemented in a custom Python-based screening pipeline across full-length protein sequences. Detected motifs were classified into three categories following previously established criteria (Kirchner et al., 2019; Schnebert et al., 2022): (i) canonical motifs, in which all required residue classes were directly encoded in the primary amino acid sequence; (ii) phosphorylation-dependent motifs, in which predicted phosphorylation of serine, threonine or tyrosine residues could generate a negatively charged residue compatible with KFERQ-like motif formation; and (iii) acetylation-dependent motifs, in which lysine acetylation was predicted to generate motif-compatible residue configurations. Overlapping pentapeptide motifs were retained as independent motif occurrences where distinct residue combinations were present. For prevalence analyses, proteins containing one or more KFERQ-like motifs were counted once per protein. Total motif abundance analyses incorporated all detected motif occurrences across each proteome. Because phosphorylation- and acetylation-dependent motifs were inferred computationally from sequence composition, these classifications represent predicted motif-generating potential rather than experimentally validated post-translational modifications.

## Results

### *P. nicotianae* releases structurally heterogeneous EVs

**Figure 1:**
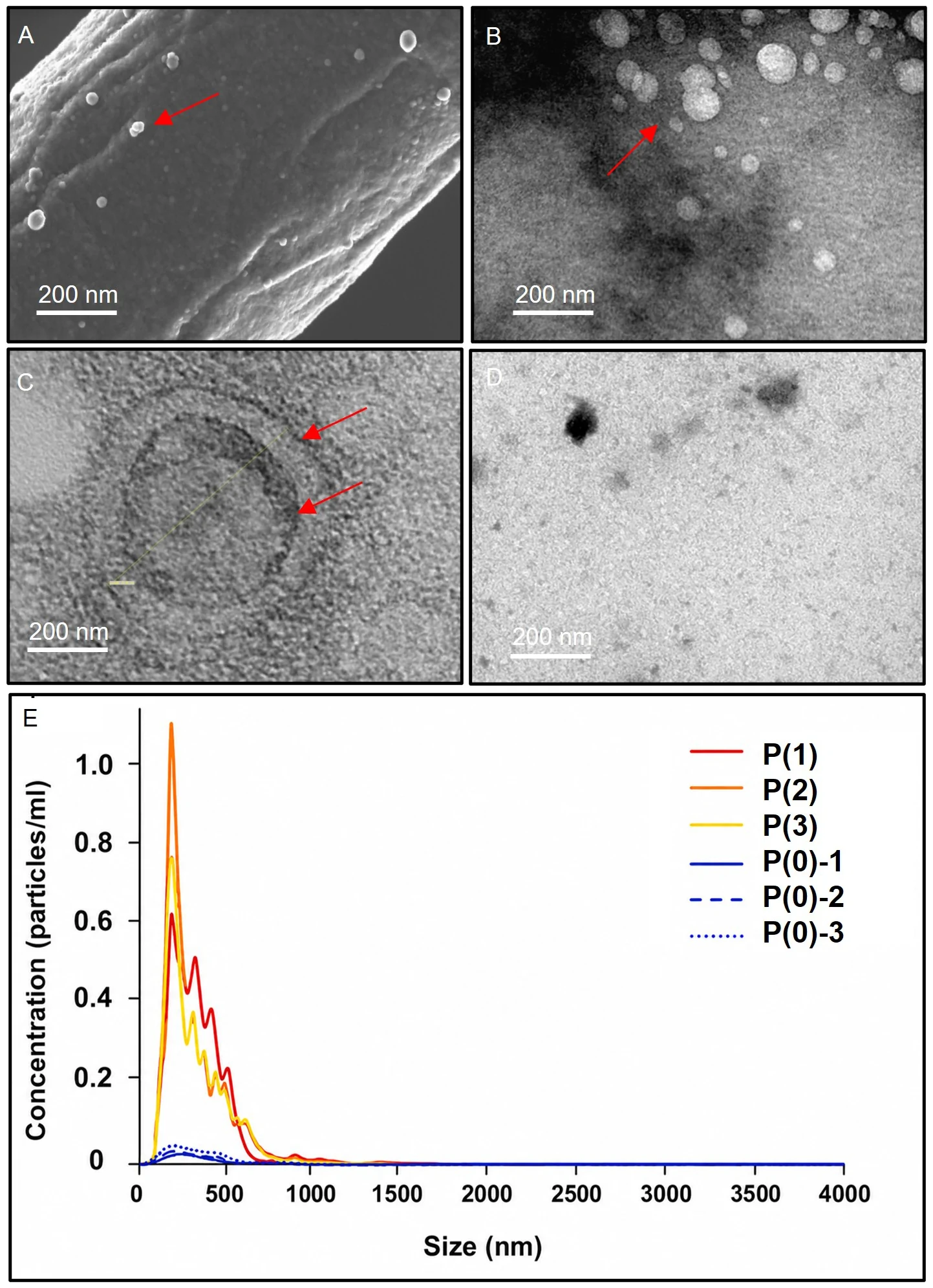
Extracellular vesicles released by *P. nicotianae*. **(A)** Scanning electron micrograph of a *P. nicotianae* hyphal cell showing discrete vesicular structures at the cell surface (arrow). **(B)** Transmission electron micrograph of extracellular particles isolated from culture medium, showing membrane-bound vesicles. **(C)** Transmission electron micrograph of an isolated vesicle displaying a double-membrane profile in cross-section (arrows indicate membrane boundaries). **(D)** TEM of the uninoculated PDB mock control. **(E)** Nanoparticle tracking analysis showing particle size distributions for three independent biological replicates of the *P. nicotianae* EV preparations (P1-P3) and uninoculated PDB mock (P(0)-1-3). Scale bars: 200 nm in **(A–D)**.

To investigate the presence of EV-like structures associated with *P. nicotianae* hyphae, mycelia cultured in liquid medium were examined using SEM. Microscopic analysis revealed discrete vesicle-like structures distributed along the hyphal surface. Some particles appeared closely associated with the cell wall, while others exhibited morphologies resembling outward membrane protrusions. Although these observations are consistent with the presence of vesicle-like structures, SEM alone cannot definitively establish their identity as extracellular vesicles (**Figure 1A**). To further characterize the extracellular particles and obtain complementary morphological evidence, particles isolated from the culture medium were examined using TEM. Membrane-bound vesicles were readily identified as discrete, spherical entities distinct from background debris (**Figure 1B**). High-magnification TEM imaging confirmed structural heterogeneity within the population, identifying both single-membrane vesicles and those displaying a distinct double-membrane bilayer (**Figure 1C**). Uninoculated PDB mock processed in parallel using the identical EV isolation protocol did not yield detectable vesicular-like structures (**Figure 1D**).

The physical properties of the isolated fractions were subsequently quantified using NTA across three independent biological replicates. The analysis demonstrated a reproducible, polydisperse size distribution, with an average particle concentration of 1.14 × 10^9^ particles/mL. The particle population was primarily distributed between 150 and 500 nm (**Figure 1E**). Across the biological replicates, the particles exhibited a modal diameter of approximately 187 nm and a mean diameter of 286 nm. In contrast, the uninoculated PDB mock control showed only low levels of background particulate material across all three replicates and did not exhibit a prominent particle population comparable to that detected in the *P. nicotianae* EV preparations (**Figure 1E**). The consistently low particle abundance in the mock controls, relative to the biological samples, indicates that the predominant particle population detected in the *P. nicotianae* preparations was associated with the biological culture rather than arising from the culture medium. Taken together, these ultrastructural observations and physical measurements are consistent with the secretion of a heterogeneous population of EVs by *P. nicotianae*.

### *P. nicotianae * EVs are associated with tissue damage and defense-related responses in *N. benthamiana*

**Figure 2:**
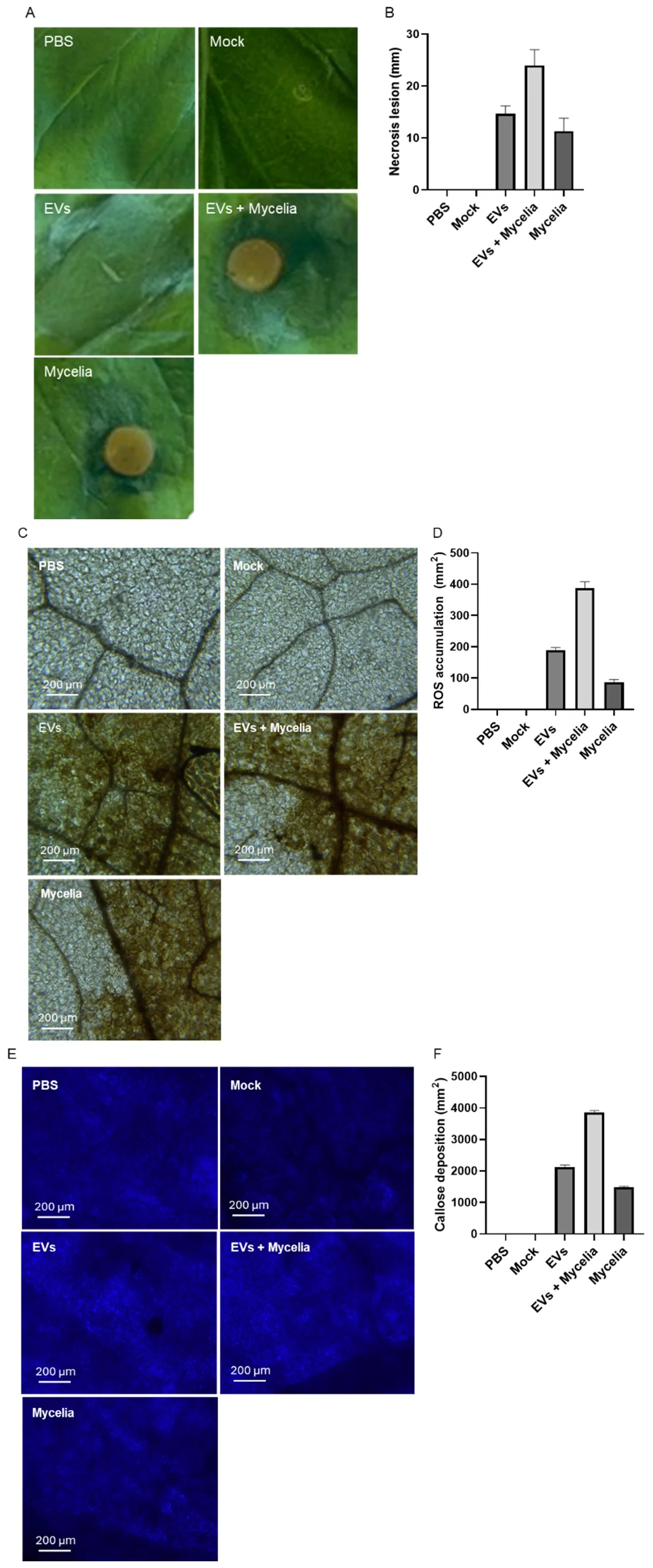
Effects of *P. nicotianae* EVs on lesion development, ROS accumulation, and callose deposition in *N. benthamiana* leaves. **(A)** Representative *N. benthamiana* leaves treated with five conditions: PBS, uninoculated PDB mock, EVs, EVs + *P. nicotianae* mycelia, and mycelia alone. **(B)** Quantification of lesion diameter following treatment with the indicated conditions. **(C)** Representative images of ROS accumulation detected by DAB staining. Brown DAB precipitates indicate sites of ROS accumulation (scale bars 200 µm). **(D)** Quantification of DAB staining intensity using ImageJ. **(E)** Representative fluorescence microscopy images showing callose deposition following aniline blue staining (scale bars 200 µm). White fluorescent spots indicate callose deposits. **(F)** Quantification of callose fluorescence intensity using ImageJ. Three independent biological replicates were analyzed, with four infiltrated leaves per treatment and five randomly selected fields of view analyzed per leaf for ROS and callose quantification. Data are presented as mean ± SD. Statistical significance was determined using one-way ANOVA followed by Tukey’s multiple comparison test, with *P* < 0.05 considered statistically significant.

To evaluate the impact of isolated EVs on host tissue integrity, *N. benthamiana* leaves were infiltrated with PBS, uninoculated PDB mock, EVs, mycelia, or a combination of EVs and mycelia (**Figure 2A**). After three days, leaves treated with PBS and uninoculated PDB mock leaves remained healthy and asymptomatic, whereas EV-treated leaves developed visible necrotic lesions comparable to those induced by mycelial treatment. The most extensive necrosis occurred in leaves receiving the combined treatment of EVs and mycelia (**Figure 2A**). Quantitative analysis of lesion diameters further validated these results, showing significantly larger areas of damage in EV, EV + mycelia and mycelia-treated tissues compared to both negative controls (**Figure 2B**; P < 0.05).

To determine whether EVs influence defense-associated responses, including accumulation and cell wall-associated responses, leaves were infiltrated with PBS, uninoculated PDB mock, EVs, mycelia and EVs + mycelia and sampled at 24 hours post-treatment. DAB staining, which detects hydrogen peroxide accumulation, revealed stronger staining in EV, mycelia, EVs + mycelia treated tissues compared to PBS and uninoculated PDB mock controls (**Figure 2C**). Quantification of staining intensity demonstrated that ROS accumulation was highest in leaves treated with EVs + mycelia, followed by EVs and mycelia alone (**Figure 2D**; P < 0.05). Similarly, aniline blue staining, used to visualize callose deposition, showed enhanced callose accumulation in leaves treated with EVs, mycelia, EVs + mycelia relative to controls (**Figure 2E**). This was further supported by quantitative analysis, which confirmed greater callose deposition in leaves treated with EVs + mycelia, followed by EVs and mycelia (**Figure 2F**; P < 0.05). Taken together, these observations show that the EV fraction is associated with both tissue damage and defense-related responses in *N. benthamiana*.

### *P. nicotianae* EVs inhibit growth of selected soil-associated bacterial isolates

**Figure 3:**
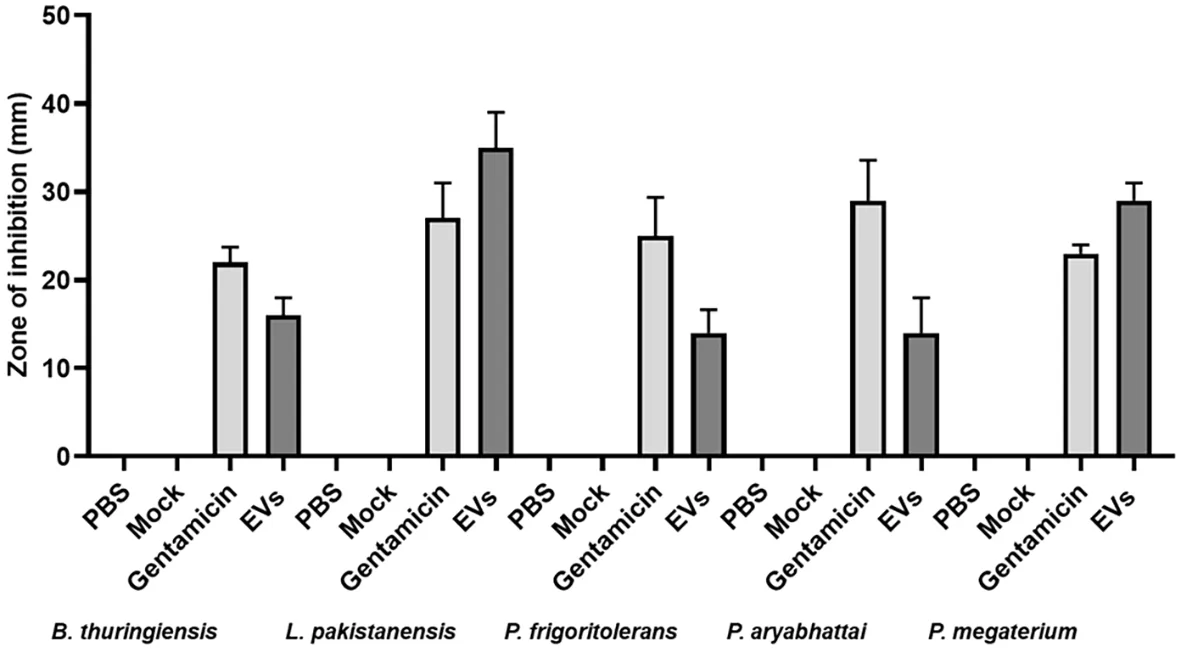
Antibacterial activity of *P. nicotianae* EVs against five soil-associated bacterial isolates: *B. thuringiensis*, *L. pakistanensis*, *P. frigoritolerans*, and *P. aryabhattai*, *P. megaterium*. PBS and uninoculated PDB mock were used as negative controls, while gentamicin was used as the positive control. Antibacterial activity was assessed based on the diameter of the inhibition zones surrounding the wells and is expressed as mean zone of inhibition (mm). Experiments were performed using three independent biological replicates, with four technical replicates per treatment. Data are presented as mean ± SD.

To determine whether EVs from *P. nicotianae* possess antimicrobial activity, the EVs were evaluated against soil-associated bacteria isolated from a crop rotation. The evaluation was conducted using an agar diffusion assay against target strains, including *B. thuringiensis*, *L. pakistanensis*, *P. frigoritolerans*, *P. aryabhattai*, and *P. megaterium*. PBS and uninoculated PDB mock were used as negative controls; gentamicin was included as a positive control to provide a benchmark for antimicrobial efficacy. On the plates inoculated with PBS and uninoculated PDB mock, there were no zones of inhibition. In contrast, the EV fraction inhibited the growth of all tested isolates, although the degree of susceptibility varied across bacterial species (**Figure 3**). Notably, for some isolates, EV-mediated inhibition exceeded that of the Gentamicin control. *L. pakistanensis* exhibited the highest sensitivity to EVs, with an inhibition zone of approximately 35 mm compared to 27 mm for Gentamicin. Similarly, *P. megaterium* showed a larger inhibition zone in response to EVs (29 mm) than to Gentamicin (23 mm). In other cases, Gentamicin remained more effective, although the EV fraction still showed measurable activity. For *B. thuringiensis*, EVs produced an inhibition zone of 16 mm compared to 22 mm for Gentamicin, while *P. frigoritolerans* showed zones of 14 mm and 25 mm, respectively. For *P. aryabhattai*, EV-mediated inhibition was approximately half that of the control, with an inhibition zone of 14 mm compared with 29 mm. These observations indicate that the EV fraction contains antibacterial activity against the bacterial isolates tested.

### The *P. nicotianae* EV proteome is dominated by catalytic, binding and transporter-associated proteins

**Figure 4:**
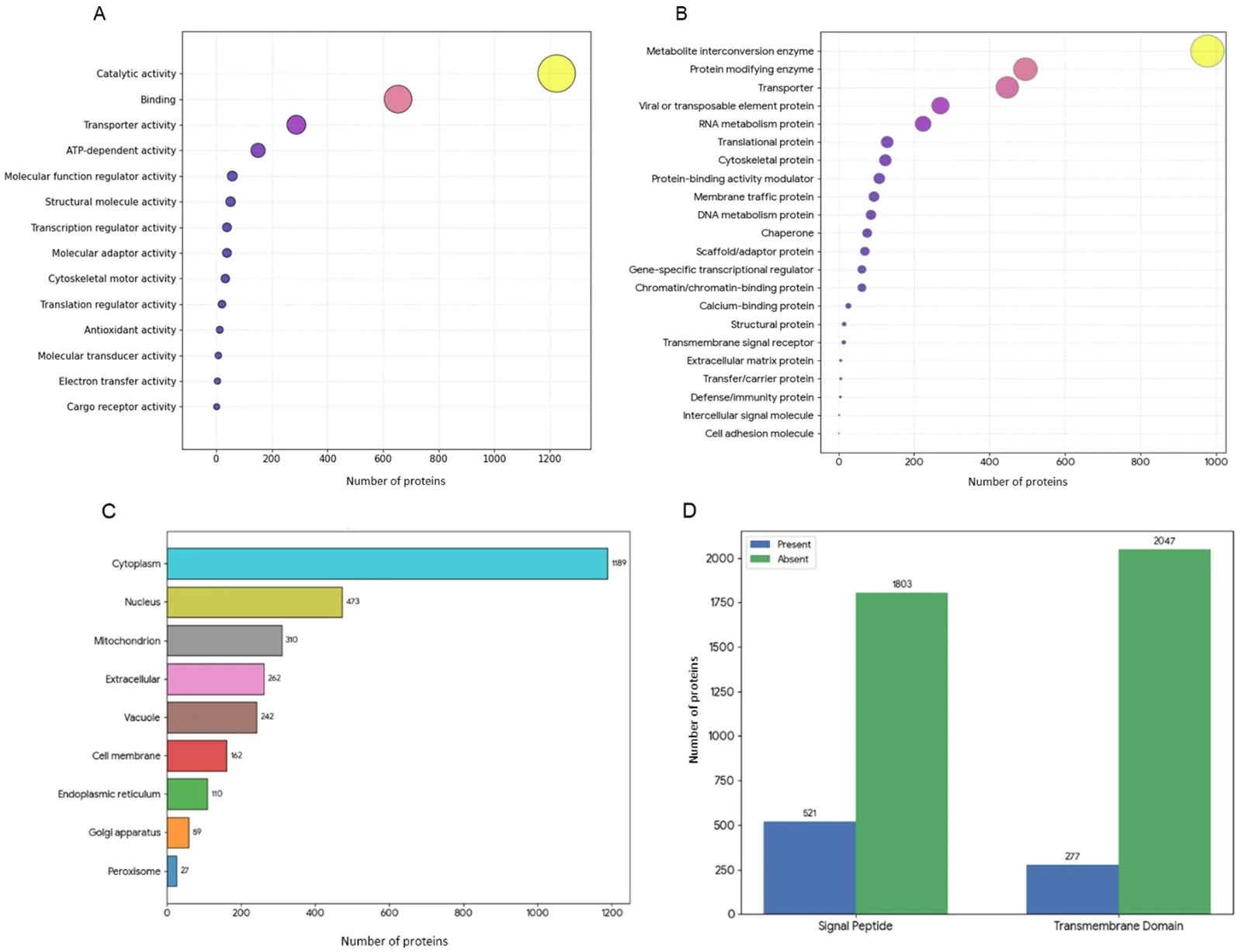
Proteomic analysis of EV proteins from *P. nicotianae*. **(A)** Molecular function of EV-associated proteins. **(B)** Protein classification of EV the proteins. **(C)** Predicted subcellular localization of EV proteins. **(D)** Prediction of signal peptides and transmembrane domains in EV-associated proteins. The green bars indicate presence and the blue bars indicate absence.

To determine the protein cargo of the *P. nicotianae* EV fraction, isolated EVs were analyzed using DIA label-free proteomics, resulting in the identification of 2,324 proteins. Gene Ontology molecular function annotation classified these proteins into 14 categories, revealing a highly diverse functional repertoire (**Figure 4A**). The major categories included catalytic activity, binding, transporter activity, ATP-dependent activity, molecular function regulator activity, structural molecule activity, and transcription regulator activity. Additional categories comprised molecular adaptor activity, cytoskeletal motor activity, translation regulator activity, antioxidant activity, molecular transducer activity, electron transfer activity, and cargo receptor activity. Catalytic activity was the most abundant category (47%), followed by binding (25.4%) and transporter activity (11.2%). This broad distribution suggests that EV cargo is not functionally specialized but instead represents a wide range of molecular activities.

Protein class annotation further resolved the EV proteome into 22 functional groups, highlighting enrichment in specific protein types (**Figure 4B**). Metabolite interconversion enzymes constituted the largest class, followed by protein-modifying enzymes and transporters. The proteome also included transposable element-associated proteins and RNA metabolism proteins. Other functional groups comprised translational proteins, cytoskeletal proteins, protein-binding activity modulators, membrane trafficking proteins, and DNA metabolism proteins. In addition, chaperones, scaffold/adaptor proteins, gene-specific transcriptional regulators, and chromatin-binding proteins were identified. The remaining categories included calcium-binding proteins, structural proteins, transmembrane signal receptors, extracellular matrix proteins, and transfer/carrier proteins, as well as proteins involved in defense, immunity, and intercellular signaling. Overall, the EV fraction contains a complex protein repertoire dominated by enzymes, interaction-associated proteins, and transport-related proteins.

Predicted subcellular localization assigned EV-associated proteins to nine cellular compartments (**Figure 4C**), including the cytoplasm, nucleus, mitochondrion, cell membrane, endoplasmic reticulum, vacuole, extracellular space, and peroxisome. Cytoplasmic proteins were the most abundant, followed by those localized to the nucleus, mitochondrion, and extracellular space. This distribution indicates that the EV proteome is not restricted to proteins with predicted extracellular localization Consistent with this, prediction of secretion-associated features showed that most EV-associated proteins lacked transmembrane domains and canonical signal peptides (**Figure 4D**). Of the 2,324 proteins identified, 277 contained predicted transmembrane domains. Similarly, 521 proteins contained predicted signal peptides. Together, these findings indicate that the *P. nicotianae* EV fraction comprises both classically secreted proteins and a larger proportion of proteins that likely follow non-canonical secretion pathways.

### EV cargo includes cell wall-degrading enzymes, elicitor-like proteins, effectors and antimicrobial proteins

We next examined whether the *P. nicotianae* EV proteome contained proteins associated with virulence. In total, 105 virulence-related proteins were identified within the EV fraction, encompassing CWDEs, elicitor-like proteins, apoplastic proteins, candidate cytoplasmic effectors and thaumatin-like proteins. These proteins ranged in length from 87 to 2,228 amino acids (**Table 1**). Cell wall-degrading enzymes constituted the largest group (51 proteins). These included glucan endo-1,3-β-D-glucosidases, 1,3-β-glucanosyltransferases, 1,3-β-glucan synthases, glycoside hydrolases, carbohydrate-binding proteins, choline dehydrogenases, pectin lyases, pectin esterases, endo-polygalacturonases, and pectate lyases. Of these, 38 contained predicted signal peptides. The EV cargo also included 22 elicitor-like proteins, such as elicitins, elicitin-like proteins, and transglutaminase elicitors, of which 18 contained predicted signal peptides. The presence of these proteins is consistent with the defense-associated responses observed following EV infiltration. However, individual elicitors were not tested separately in this study, and their specific contributions to cell death, ROS accumulation, or callose deposition remain to be experimentally determined. Apoplastic protein classes (20 proteins) were also identified, including cystatin-domain proteins, small cysteine-rich proteins, SCP-domain proteins, and Kazal-like domain-containing proteins. Among these, 18 contained predicted signal peptides. In addition, 12 candidate cytoplasmic effectors were detected, including RxLR and Crinkler (CRN) proteins. Of the RxLR effectors, nine contained predicted signal peptides, while both identified CRN proteins lacked predicted signal peptides. The detection of these effectors in the EV fraction supports the hypothesis that EVs may represent an alternative export route for virulence-associated proteins. However, direct delivery into host cells was not experimentally assessed and should not be inferred from proteomic data alone. The EV proteome further included three thaumatin-like proteins, a class associated with antimicrobial activity in both plant and pathogen systems; all contained predicted signal peptides. Their presence is consistent with the antibacterial activity observed in agar diffusion assays, although the specific proteins responsible for this effect remain unidentified. Collectively, these findings indicate that the *P. nicotianae* EV fraction contains a structured repertoire of virulence-associated proteins, comprising degradative enzymes, elicitor-like proteins, apoplastic proteins, candidate cytoplasmic effectors, and proteins with potential antimicrobial activity.

**Table 1:** The virulence factors identified in *P. nicotianae* EVs.

Gene ID	Protein description	Protein length	Signal peptide	Functional category
PPTG_24766	Glucan endo-1,3-beta-D-glucosidase	654	SP	CWDE
PPTG_18983	Glucan endo-1,3-beta-D-glucosidase	443	SP	CWDE
PPTG_17873	Glucan endo-1,3-beta-D-glucosidase	1084	SP	CWDE
PPTG_17498	Glucan endo-1,3-beta-D-glucosidase	579	SP	CWDE
PPTG_17188	Glucan endo-1,3-beta-D-glucosidase	426	No	CWDE
PPTG_17187	Glucan endo-1,3-beta-D-glucosidase	458	SP	CWDE
PPTG_17201	Glucan endo-1,3-beta-D-glucosidase	287	SP	CWDE
PPTG_16550	Glucan endo-1,3-beta-D-glucosidase	415	SP	CWDE
PPTG_16243	Glucan 1,3-beta-glucosidase	454	SP	CWDE
PPTG_15563	Glucan endo-1,3-beta-D-glucosidase	384	SP	CWDE
PPTG_13594	Glucan endo-1,3-beta-D-glucosidase	87	No	CWDE
PPTG_11267	Glucan endo-1,3-beta-D-glucosidase	292	SP	CWDE
PPTG_09844	1,3-beta-glucanosyltransferase	502	SP	CWDE
PPTG_09868	Glucan endo-1,3-beta-D-glucosidase	824	No	CWDE
PPTG_10954	Glucan endo-1,3-beta-D-glucosidase	394	SP	CWDE
PPTG_22560	Glucan endo-1,3-beta-D-glucosidase	351	SP	CWDE
PPTG_08579	1,3-beta-glucan synthase	2228	No	CWDE
PPTG_03836	Glucan endo-1,3-beta-D-glucosidase	556	SP	CWDE
PPTG_01483	Glucan 1,3-beta-glucosidase	373	SP	CWDE
PPTG_02889	Glucan endo-1,3-beta-D-glucosidase	586	No	CWDE
PPTG_14175	GH18 domain-containing protein	383	No	CWDE
PPTG_12451	GH16 domain-containing protein	726	SP	CWDE
PPTG_12473	GH16 domain-containing protein	758	SP	CWDE
PPTG_12475	GH16 domain-containing protein	731	SP	CWDE
PPTG_15925	Choline dehydrogenase	587	No	CWDE
PPTG_12009	Glycoside hydrolase	573	SP	CWDE
PPTG_18765	Glycoside hydrolase family 5 domain-containing protein	638	No	CWDE
PPTG_17668	Glycoside hydrolase family 19 catalytic domain-containing protein	229	SP	CWDE
PPTG_15981	Glycoside hydrolase	279	No	CWDE
PPTG_09835	Glycoside hydrolase family 5 domain-containing protein	494	SP	CWDE
PPTG_10229	Glycoside hydrolase 131 catalytic N-terminal domain-containing protein	370	SP	CWDE
PPTG_06145	Glycoside hydrolase family 5 domain-containing protein	371	No	CWDE
PPTG_05788	Glycoside hydrolase family 5 domain-containing protein	322	No	CWDE
PPTG_05786	Glycoside hydrolase family 5 domain-containing protein	645	No	CWDE
PPTG_03844	Glycoside hydrolase family 5 domain-containing protein	582	No	CWDE
PPTG_02086	Carbohydrate-binding protein	200	SP	CWDE
PPTG_11794	Carbohydrate-binding module family 19 domain-containing protein	312	SP	CWDE
PPTG_17499	Pectin lyase	465	SP	CWDE
PPTG_15712	Pectin lyase	376	SP	CWDE
PPTG_12891	Pectin lyase	439	SP	CWDE
PPTG_10338	Pectin esterase	359	SP	CWDE
PPTG_15179	Endo-polygalacturonase	361	SP	CWDE
PPTG_15163	Endo-polygalacturonase	391	SP	CWDE
PPTG_13804	Endo-polygalacturonase	512	SP	CWDE
PPTG_11094	Probable pectate lyase F	252	SP	CWDE
PPTG_11095	Probable pectate lyase F	249	SP	CWDE
PPTG_03562	Probable pectate lyase F	261	SP	CWDE
PPTG_08662	Pectate lyase	206	SP	CWDE
PPTG_07724	Glucan endo-1,3-beta-D-glucosidase	298	SP	CWDE
PPTG_17779	Elicitin	260	No	PAMP
PPTG_15237	Elicitin	160	SP	PAMP
PPTG_13821	Elicitin	181	SP	PAMP
PPTG_13458	Elicitin-like protein	209	SP	PAMP
PPTG_12447	Elicitin	191	SP	PAMP
PPTG_12310	Elicitin	377	SP	PAMP
PPTG_12290	Elicitin	145	SP	PAMP
PPTG_12308	Elicitin	387	No	PAMP
PPTG_12305	Elicitin	310	SP	PAMP
PPTG_11410	Elicitin	142	SP	PAMP
PPTG_10345	Elicitin	364	No	PAMP
PPTG_09075	Elicitin	118	SP	PAMP
PPTG_09080	Elicitin	183	SP	PAMP
PPTG_08950	Elicitin	184	SP	PAMP
PPTG_09073	Elicitin	120	SP	PAMP
PPTG_07488	Elicitin-like protein	193	SP	PAMP
PPTG_05250	Elicitin-like protein	235	SP	PAMP
PPTG_16233	Transglutaminase elicitor	910	SP	PAMP
PPTG_19674	Transglutaminase elicitor (Fragment)	579	No	PAMP
PPTG_16236	Transglutaminase-like domain-containing protein	530	SP	PAMP
PPTG_16234	Transglutaminase elicitor	768	SP	PAMP
PPTG_18856	Cystatin domain-containing protein	280	SP	Apoplastic
PPTG_03490	Cystatin domain-containing protein	146	No	Apoplastic
PPTG_03489	Cystatin domain-containing protein	126	SP	Apoplastic
PPTG_01934	Small cysteine rich protein SCR108	110	SP	Apoplastic
PPTG_15512	SCP domain-containing protein	443	SP	Apoplastic
PPTG_14103	SCP2 domain-containing protein	249	No	Apoplastic
PPTG_11091	SCP domain-containing protein	239	SP	Apoplastic
PPTG_08721	SCP domain-containing protein	294	SP	Apoplastic
PPTG_08720	SCP domain-containing protein	260	SP	Apoplastic
PPTG_08738	SCP domain-containing protein	173	SP	Apoplastic
PPTG_02297	SCP domain-containing protein	152	SP	Apoplastic
PPTG_02299	SCP domain-containing protein	164	SP	Apoplastic
PPTG_00482	SCP domain-containing protein	167	SP	Apoplastic
PPTG_01148	SCP domain-containing protein	295	SP	Apoplastic
PPTG_18808	Kazal-like domain-containing protein	124	SP	Apoplastic
PPTG_04341	Kazal-like domain-containing protein	459	SP	Apoplastic
PPTG_03107	Kazal-like domain-containing protein	350	SP	Apoplastic
PPTG_03106	Kazal-like domain-containing protein	138	SP	Apoplastic
PPTG_02604	Kazal-like domain-containing protein	329	SP	Apoplastic
PPTG_08697	Crinkler effector protein N-terminal domain-containing protein	433	No	Apoplastic
PPTG_05866	Crinkler effector protein N-terminal domain-containing protein	442	No	Cytoplasmic
PPTG_19007	RxLR effector protein	361	SP	Cytoplasmic
PPTG_17015	RxLR effector protein	161	SP	Cytoplasmic
PPTG_17122	RxLR effector protein	204	SP	Cytoplasmic
PPTG_16462	RxLR effector protein	180	SP	Cytoplasmic
PPTG_15318	RxLR effector PexRD54 WY domain-containing protein	315	No	Cytoplasmic
PPTG_14579	RxLR effector protein	141	SP	Cytoplasmic
PPTG_14580	RxLR effector protein	139	SP	Cytoplasmic
PPTG_07169	RxLR effector protein	173	SP	Cytoplasmic
PPTG_02753	RxLR effector protein	211	SP	Cytoplasmic
PPTG_02751	RxLR effector protein	286	SP	Cytoplasmic
PPTG_10199	Thaumatin-like protein	297	SP	Antimicrobial
PPTG_08708	Thaumatin-like protein	384	SP	Antimicrobial
PPTG_17477	Thaumatin-like protein	429	SP	Antimicrobial

### Comparative proteomics reveals shared EV-associated protein categories and lineage-specific variation

**Figure 5:**
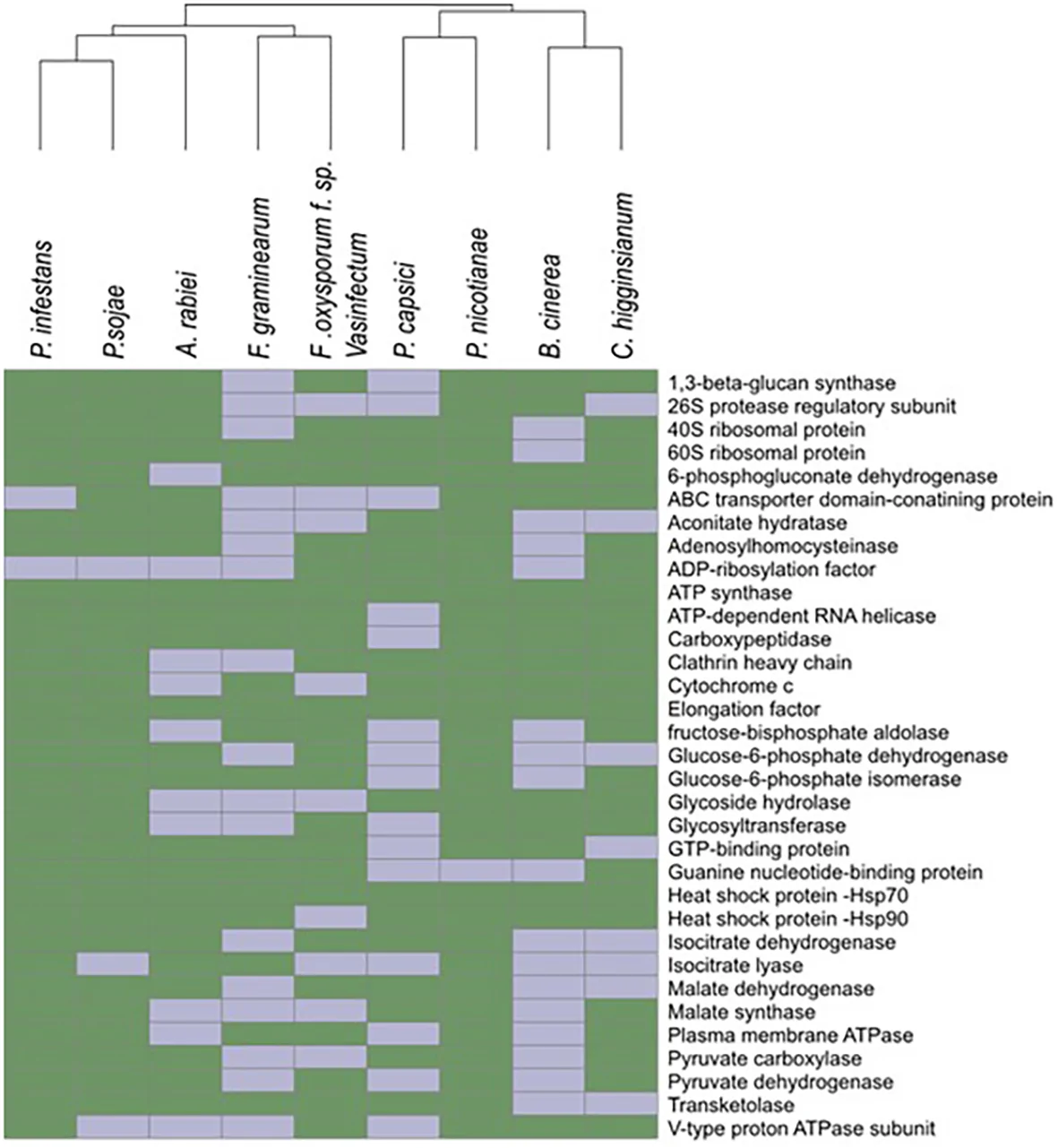
Comparative distribution of EV-associated protein categories across filamentous pathogens. Heatmap showing the presence-absence profiles of homologous EV-associated proteins identified across nine filamentous pathogen species, including the fungi *F. oxysporum f.* sp. *vasinfectum*, *F. graminearum*, *C. higginsianum*, *B. cinerea* and *A. rabiei*, and the oomycetes *P. capsici*, *P. sojae*, *P. infestans* and *P. nicotianae*. Rows represent annotated protein homologues and columns represent individual species. Protein occurrence was scored using binary presence-absence data derived from published EV proteomes. Green indicates protein presence and purple indicates protein absence. EV-associated protein categories shared across multiple taxa included ATP synthase subunits, elongation factors, and Hsp70, whereas other protein categories displayed variable or lineage-restricted distributions.

To assess whether the *P. nicotianae* EV proteome shares common features with other filamentous pathogens, its protein composition was compared with published EV proteomes from the fungi *F. oxysporum f.* sp. *vasinfectum*, *F. graminearum*, *C. higginsianum*, *B. cinerea*, and *A. rabiei*, and the oomycetes *P. capsici*, *P. sojae*, and *P. infestans* (**Figure 5**). Comparisons were based on manually curated and standardized EV-associated protein categories derived from the protein annotations reported in the published datasets. A binary presence-absence matrix was generated for each standardized protein category across all species and used to calculate Jaccard similarity coefficients and visualize the distribution of EV-associated protein categories (**Figure 5**).

A total of 32 protein categories were identified across the EV proteomes of the nine species analyzed. Of these, 3 protein categories, comprising ATP synthase subunits, elongation factors, and Hsp70, were detected in all species, representing a shared EV-associated protein categories. An additional 12 protein categories were present in seven or eight species, including 1,3-β-glucan synthase, adenosylhomocysteinase, clathrin heavy chain, glucose-6-phosphate isomerase, GTP-binding proteins, transketolase, 40S and 60S ribosomal proteins, 6-phosphogluconate dehydrogenase, ATP-dependent RNA helicase, carboxypeptidase, and heat shock protein 90 (Hsp90). A further 15 protein categories were detected in five or six species, indicating moderate conservation across the analyzed filamentous pathogens, whereas ADP-ribosylation factor and malate synthase were identified in only four species.

Within the genus *Phytophthora*, 9 protein categories were consistently detected across all four species, including pyruvate carboxylase, malate dehydrogenase, malate synthase, isocitrate dehydrogenase, transketolase, glycoside hydrolase, clathrin heavy chain, aconitate hydratase, and adenosylhomocysteinase. Overall, the *P. nicotianae* EV proteome shared several EV-associated protein categories associated with fundamental cellular processes. Greater similarity was observed among *Phytophthora* species than between oomycetes and fungi, consistent with the presence of both shared and lineage-specific EV-associated protein categories.

### KFERQ-like motifs are prevalent in EV-associated proteins of filamentous pathogens and *Phytophthora* RxLR effectors

**Figure 6:**
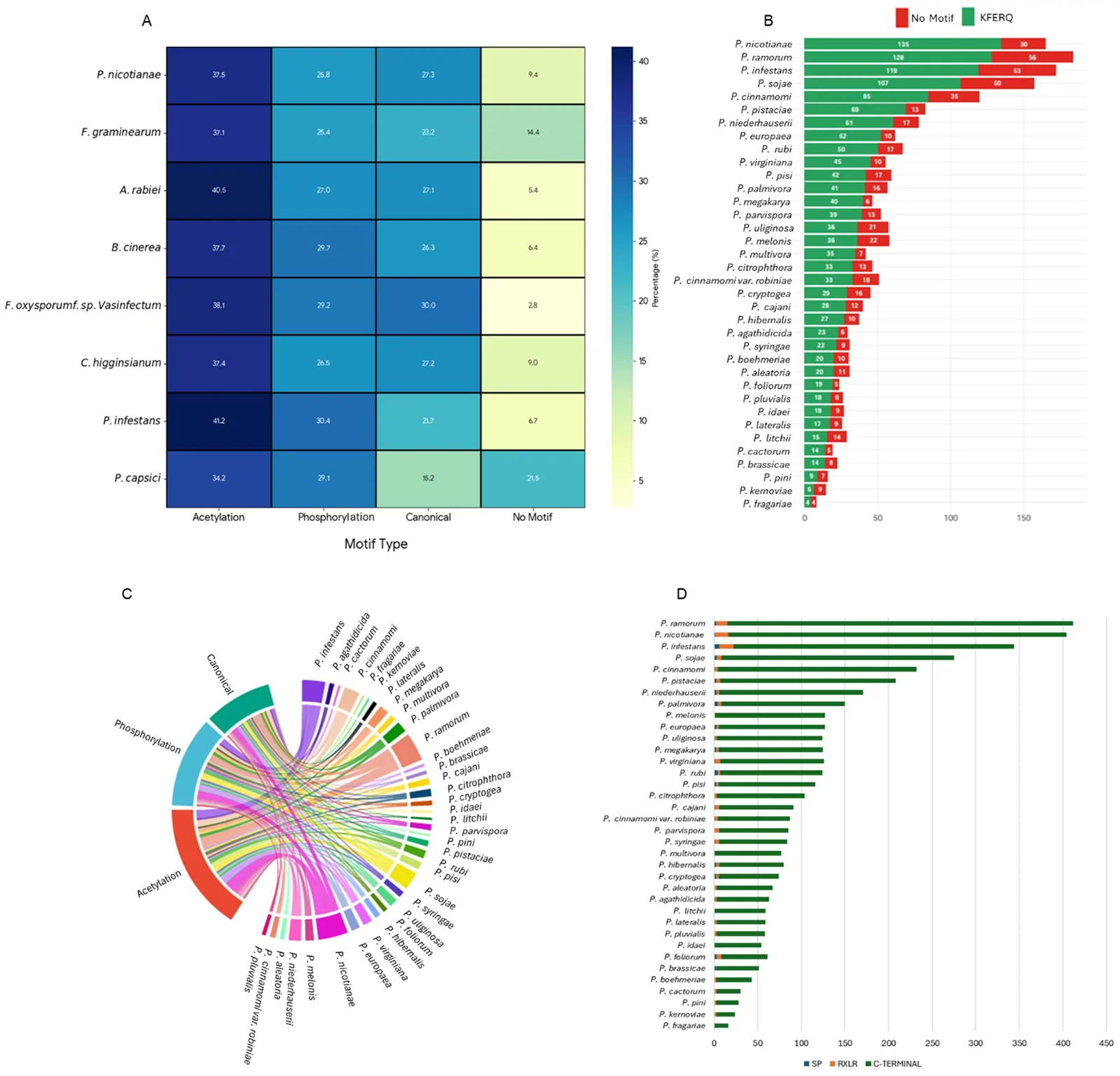
Analysis of KFERQ motifs in filamentous pathogens and *Phytophthora* RxLR effectors. **(A)** Heatmap showing the relative proportion (%) of KFERQ motif types identified in EV-associated proteins across eight filamentous pathogen species, including acetylation-, phosphorylation-, and canonical motifs, as well as proteins lacking identifiable motifs (No motif). **(B)** Color intensity reflects the percentage contribution of each motif category within each species. Analysis of KFERQ motifs in 36 *Phytophthora* RxLR effectors, where green indicates the presence and red indicates the absence of motifs. **(C)** Distribution of KFERQ motif types (acetylation, phosphorylation, and canonical) among the 36 *Phytophthora* RxLR effectors. **(D)** Positional distribution of KFERQ motifs within RxLR effector sequences, where blue indicates motifs located in the signal peptide, orange indicates the RxLR motif region, and green indicates the C-terminal region.

To assess the distribution of KFERQ-like motifs in EV-associated proteins of filamentous plant pathogens, EV proteomes from *F. oxysporum f.* sp. *vasinfectum, F. graminearum, C. higginsianum, B. cinerea, A. rabiei*, and the oomycetes *P. capsici, P. nicotianae* and *P. infestans* were screened using established physicochemical criteria for KFERQ-like motif identification. Predicted KFERQ-like motifs were detected at high frequency across all species analyzed. Among fungal pathogens, motif-containing proteins represented 97% of EV-associated proteins in *F. oxysporum f.* sp. *vasinfectum*, 95% in *A. rabiei*, 94% in *B. cinerea*, 91% in *C. higginsianum* and 85% in *F. graminearum.* Within oomycete EV proteomes, 93% of proteins from *P. infestans* and 78% from *P. capsici* contained at least one predicted KFERQ-like motif. Classification of motif categories revealed that acetylation-dependent variants predominated across all proteomes analyzed, followed by phosphorylation-dependent and canonical motifs (**Figure 6A**).

RxLR effectors, which constitute major virulence determinants in *Phytophthora* species, were subsequently examined separately within the EV proteomes of *P. infestans* and *P. nicotianae.* Predicted KFERQ-like motifs were identified in 88% of *P. infestans* RxLR effectors and 40% of *P. nicotianae* RxLR effectors detected within EV-associated datasets. As phosphorylation- and acetylation-dependent motifs were inferred computationally from sequence composition, these classifications reflect predicted motif-generating potential rather than experimentally validated post-translational modifications. Collectively, these analyses demonstrate that KFERQ-like motifs are broadly represented within EV-associated protein repertoires of filamentous plant pathogens.

To determine whether this pattern extended more broadly across conserved effector repertoires, conserved RxLR effectors from 36 *Phytophthora* species (Salasini et al., 2026) were analyzed using the same computational framework. Predicted KFERQ-like motifs were detected in 40-95% of RxLR effectors across all species examined (**Figure 6B**). In 35 of the 36 species analyzed, motif-containing effectors outnumbered those lacking predicted motifs, with *Phytophthora kernoviae* exhibiting the lowest proportion (~40%). Similar to the patterns observed within EV-associated proteomes, acetylation- and phosphorylation-dependent motifs were consistently more abundant than canonical motifs across the majority of species analyzed (**Figure 6C**).

Positional mapping across RxLR protein architectures showed that predicted KFERQ-like motifs occurred throughout the signal peptide, RxLR domain and C-terminal effector region, with the greatest absolute frequency observed within C-terminal regions (**Figure 6D**). Given that the C-terminal region constitutes the largest structural component of most RxLR proteins and mediates the majority of host-interaction activities, motif distribution was interpreted relative to overall regional representation within the protein architecture. Together, these findings suggest that predicted KFERQ-like motifs are widely distributed across EV-associated proteins and conserved RxLR effector repertoires in filamentous plant pathogens and *Phytophthora* species.

## Discussion

Extracellular vesicles have emerged as important mediators of virulence, intercellular communication, and host modulation in diverse bacterial and mammalian systems (Yoon et al., 2014; Kim et al., 2016; Bose et al., 2021; Pirolli et al., 2021; Munoz et al., 2022). However, EV production and functional roles remain comparatively unexplored in plant-pathogenic oomycetes. In this study, we isolated and characterized EVs from *P. nicotianae* cultures and investigated their biological activity and protein composition. The integration of morphological, biochemical, and proteomic approaches provides evidence for the presence of a heterogeneous EV population associated with *P. nicotianae* cultures and provides insights into their potential roles during host interaction and microbial competition.

Scanning electron microscopy revealed vesicle-like structures associated with the hyphal surface of *P. nicotianae*. Although SEM provides evidence of extracellular vesicle-like structures, this approach alone cannot distinguish EVs from other extracellular components, such as matrix-associated material. Therefore, SEM observations were interpreted together with TEM and NTA. TEM confirmed the presence of membrane-bound vesicles with both single- and double-membrane morphologies, while NTA demonstrated a reproducible and heterogeneous particle population. Together, these complementary approaches support the presence of EVs within *P. nicotianae* culture filtrates and highlight the structural diversity of vesicles produced by this pathogen.

The biological activity of the isolated EV fraction was further demonstrated through functional assays. Application of the EV fraction to *N. benthamiana* leaves induced necrotic lesions, ROS accumulation, and callose deposition, indicating activation of plant defense-associated responses. Moreover, co-inoculation of the EV fraction with *P. nicotianae* mycelia resulted in enhanced necrosis, ROS accumulation, and callose deposition compared with mycelial inoculation alone. These findings indicate that the EV fraction contains biologically active components capable of influencing plant responses and may contribute to *P. nicotianae*-plant interactions. Whether EVs directly enhance pathogen colonization or contribute to infection processes during natural host interactions remains an important area for future investigation. In addition, the EV fraction inhibited the growth of all bacterial isolates tested, indicating that *P. nicotianae* EVs may also influence microbial interactions. The contribution of EV-associated molecules, including specific cargo components, to this antibacterial activity remains to be elucidated.

Proteomic analysis identified 2,324 proteins associated with the isolated EV fraction. Gene Ontology and protein class analyses revealed enrichment of proteins involved in catalytic activity, transport, metabolism, and cellular processes. Similar functional categories have been reported in EV proteomes from other filamentous pathogens, suggesting the presence of shared EV-associated components that support fundamental cellular functions across fungi and oomycetes (Garcia-Ceron et al., 2021; Fang et al., 2021; Rutter et al., 2022; De Vallée et al., 2023; Zhu et al., 2023; Breen et al., 2025; Ghaheri et al., 2025).

In addition to shared cellular proteins, several proteins previously associated with pathogenicity, including cell wall-degrading enzymes CWDEs, PAMPs, and RxLR and CRN effectors, were detected in the EV fraction. Their presence suggests that EVs may carry molecules with potential roles in host-pathogen interactions. The EV proteome also contained proteins with predicted antimicrobial functions, including thaumatin-like proteins. Although these findings suggest that EV-associated proteins may contribute to interactions with host cells and other microorganisms, the functional contribution of individual proteins remains to be experimentally established.

Comparative proteomic analysis further demonstrated that many EV-associated protein categories identified in *P. nicotianae* were shared among filamentous pathogens, particularly proteins involved in metabolism, transport, and stress responses. Greater conservation was observed among *Phytophthora* species compared with comparisons between oomycetes and fungi, indicating that EV composition may reflect evolutionary relationships.

Among the shared EV-associated protein categories, HSP70 was consistently represented across the analyzed EV proteomes. In mammalian systems, HSP70 has been implicated in cargo loading through the recognition of proteins containing KFERQ-like motifs (Ferreira et al., 2022; Xu et al., 2025). Motivated by these observations, we performed a computational analysis of KFERQ-like motifs within EV-associated proteins and identified these motifs in a large proportion of EV-associated proteins and *Phytophthora* RxLR effectors. This analysis provides a hypothesis-generating framework suggesting that KFERQ-like motifs may represent putative conserved features associated with EV cargo regulation in filamentous pathogens. However, the presence of these motifs alone does not establish HSP70-dependent recognition, selective cargo incorporation, or conservation of mammalian-like EV loading mechanisms in oomycetes. Further investigation will be required to determine whether these motifs contribute to EV cargo regulation and how they may influence cargo composition in *P. nicotianae*.

Overall, this study demonstrates that *P. nicotianae* produces structurally heterogeneous EVs with biological activity in plant and bacterial assays, highlighting their potential involvement in host responses and microbe-microbe interactions under the experimental conditions tested. The identification of diverse EV-associated proteins, including proteins associated with pathogenicity-related processes and predicted antimicrobial functions, provides new insights into the functional complexity of EV cargo in plant-pathogenic oomycetes. Comparative analysis further revealed shared EV-associated protein categories across filamentous fungi and oomycetes, alongside lineage-specific variation. Together, these findings provide a foundation for further investigation of EV biology in *P. nicotianae* and offer new perspectives on the potential contribution of EVs to host-pathogen and microbe-microbe interactions. Future studies are needed to clarify the mechanisms governing EV biogenesis and cargo incorporation, and to determine the specific contributions of individual EV-associated molecules to host and microbial interactions.

## Data Availability

The original contributions presented in this study are included in the article and are publicly available at https://doi.org/10.6084/m9.figshare.331366130. Further inquiries can be directed to the corresponding author.
